# Engaging older adults in diagnostic safety: implementing a diagnostic communication note sheet in a primary care setting

**DOI:** 10.3389/frhs.2024.1474195

**Published:** 2025-01-13

**Authors:** Alberta Tran, Leah Blackall, Mary A. Hill, William Gallagher

**Affiliations:** ^1^ MedStar Institute for Quality and Safety, MedStar Health Research Institute, Columbia, MD, United States; ^2^ Institute of Health Policy, Management, & Evaluation, University of Toronto, Toronto, ON, Canada; ^3^ Michael Garron Hospital, Toronto East Health Network, Toronto, ON, Canada; ^4^ Family Medicine Department, Georgetown University School of Medicine, Washington, DC, United States

**Keywords:** diagnostic safety, diagnostic communication, patient engagement, older adult, primary care, quality improvement

## Abstract

**Introduction:**

Adults over the age of 65 are at a higher risk for diagnostic errors due to a myriad of reasons. In primary care settings, a large contributor of diagnostic errors are breakdowns in information gathering and synthesis throughout the patient-provider encounter. Diagnostic communication interventions, such as the Agency for Healthcare Research and Quality's “Be the Expert on You” note sheet, may require adaptations to address older adults' unique needs.

**Methods:**

We recruited and partnered with older adult patients (*n* = 6) in focus group sessions to understand their perspectives on diagnostic communication and the existing AHRQ note sheet. A two-page communication and clinic workflow tool was developed and implemented over a 6-month period using three Plan-Do-Check-Act cycles. Physicians, nurses, staff, and patients were surveyed.

**Results:**

Most older adult patients (*n* = 31) found the tailored diagnostic communication note sheet to be easy-to-use, helpful for provider communication, and would recommend its use to other patients. Physicians and staff members were satisfied with the note sheet and described few challenges in using it in practice.

**Discussion:**

Our findings contribute to the growing body of evidence around diagnostic safety interventions and patient engagement by demonstrating the feasibility and benefits of actively involving older adult patients in quality initiatives.

## 1 Introduction

Diagnostic errors—or the failures to establish an accurate and timely explanation of a patient's health problem(s) and/or communicate that explanation to the patient (1)—are common, costly, and pose risk for serious patient harm (2–4). Researchers estimate that 1 in 20 primary care patients in the United States (U.S.) experience a diagnostic error each year (3), and that most patients will experience a diagnostic error in their lifetime (1). In particular, older adults, typically described as individuals 65 and older, are vulnerable to preventable harms and deaths (5) and are at a higher risk of diagnostic errors due to a myriad of reasons. A systematic review of diagnostic errors in older adults found that diagnostic errors involving older adults were common and comprised both overdiagnosis and underdiagnosis; the presence of physical comorbidities was consistently associated with lower accuracy in diagnosing several prevalent and high disease-burden conditions in this population (6). The *Advancing Diagnostic Excellence for Older Adults Workshop* led by the National Academies of Sciences, Engineering, and Medicine (NASEM) described the importance of recognizing how diagnosis can become more difficult as medical complexity increases with age; this differentiation between ongoing or chronic disease trajectories vs. new, acute issues can complicate the diagnostic process (7). Ageist stereotypes may additionally perpetuate implicit biases towards older adults, and clinicians may mistake early symptoms of disease as subtle changes to physical and mental states that are part of the aging process (7–9).

Patient-facing strategies to engage patients in clearly communicating about their symptoms and experiences may be effective tools to reduce diagnostic errors. In 2021, the Agency for Healthcare Research and Quality (AHRQ) developed its *Toolkit for Engaging Patients to Improve Diagnostic Safety* to enhance communication and information sharing within the patient-provider encounter (10). The “Be the Expert on You” note sheet– a strategy aimed to help patients organize their medical stories and concerns in preparation for their health appointments—was the product of an extensive literature review of patient-facing materials to enhance communication, built off existing examples of patient-facing resources, and was based on the Subjective, Objective, Assessment, and Plan (SOAP) note communication template to support clinicians' existing cognitive models of diagnosis (11, 12).

Existing interventions for improving diagnostic safety may require additional efforts or considerations to engage older adults and address their unique needs. For example, several studies have found that older adults are less likely to feel comfortable speaking up about problems they've experienced (13), and those with low health literacy levels affect their ability to participate optimally in healthcare (14, 15). Older adult patients may require tailored or different interventions to address their unique needs in patient safety initiatives.

Evidence suggests that tailoring interventions to specific populations can lead to improvements in health equity and healthcare outcomes (16, 17). Interventions tailored for historically marginalized racial and ethnic populations, for example, have led to improvements in chronic disease management and overcoming barriers related to access to care and cultural competency (18, 19). Barriers to engaging older adults in research and quality improvement initiatives have been identified, including the lack of established relationships with researchers, exclusion from the intervention planning stages, varying preferences for engagement, and a lack of understanding of the benefits of participation (20, 21). Additionally, older adults may experience cognitive impairments and lack of support from medical providers when participating in quality improvement efforts, which can hinder their willingness to participate (21, 22). Older adults may also be more resistant to changes, emphasizing the need to provide reassurance about the benefits of new processes or interventions (23).

Several interventions aimed at enhancing communication specifically between older adult patients and their care providers have been developed, with many of these interventions are focused on shared decision-making (24) or advance care planning (e.g., SHARING Choices) (25). However, because these interventions generally focus on processes *after* a diagnosis has been made, they are limited in improving the information gathering and history-taking aspects of clinical care to inform a diagnosis. Further, while the “Be the Expert on You” note sheet is widely available and already being used by several health organizations and health provider groups across the U.S. to improve diagnostic safety, its use specifically for the older adult population has not yet been evaluated.

In this paper, we present the adaptation and implementation of this existing AHRQ tool in a quality improvement initiative aimed at improving diagnostic communication between older adult patients and physicians in a family medicine, primary care residency clinic that serves a diverse, underserved patient population. We sought to assess whether the note sheet was an effective and efficient way to improve diagnostic communication between older adult patients and physicians. Our project aims were to (i) review and modify the existing AHRQ “Be the Expert on You” note sheet in focus group sessions with recruited older adult patients; (ii) implement the modified note sheet in practice in 3 Plan-Do-Check-Act (PDCA) phases focused on patient engagement; and (iii) evaluate the impact of the revised note sheet on patient and physician satisfaction with diagnostic communication.

## 2 Materials and methods

### 2.1 Study design

This was a one-year quality improvement project with implementation occurring in three general phases. The study team consisted of a researcher to lead study design and planning, data collection and analysis, and evaluation; a research assistant to support patient recruitment, data collection, and data analysis; and a physician champion to lead physician and staff education and practice-wide implementation. To meaningfully involve patients and end users throughout the process, the three phases included (1) focus groups with recruited older adult patients to review and modify the note sheet, (2) training and engagement with physicians, nurses, and staff, and (3) implementing the note sheet with three PDCA cycles over a 6-month period. Institutional Review Board approval was obtained from MedStar Health Research Institute/Georgetown University prior to focus group recruitment and implementation.

### 2.2 Setting and participants

Focus group recruitment and project implementation occurred in a large family practice setting located in an urban area located in the Southeastern U.S. The practice location consists of 7 attending physicians, 3 registered or licensed practical nurses, 4 medical assistants and 11 other practice staff members (e.g., front desk and administrative staff, and patient navigators). It additionally serves as a graduate medical education (GME) training site for 12 family medicine residents and serves a diverse population of patients (8,000 patient visits in 2022; 22% adults who are 65 years of age and older, 15% Hispanic, 68% Black or African American).

### 2.3 Phase 1: older adult patient focus groups to understand diagnostic communication and review AHRQ “Be the expert on you” note sheet

Patient recruitment involved the distribution of informational flyers, receipt of physician/staff referrals, and conduct of eligibility screening from daily patient appointment information from October 2022 to December 2022. Adults who were unable to provide verbal consent, non-English speakers, or those with suspected or documented impaired mental capacity were excluded. Patients were informed that they would be compensated with a $100 gift card for their complete participation in the project.

Four 60-minute focus group sessions with 6 recruited patient participants were conducted in January and February 2023. Focus groups were held in-person and at the practice location, depending on patients' preferences and communication needs. Patient focus groups were facilitated by the project lead (AT). Prior to the start of each focus group session, participants were informed about the purpose of the study, the procedures involved, and the voluntary nature of their participation. Verbal consent was obtained from all participants after a detailed explanation of the study's objectives and confidentiality measures. Participants were given the opportunity to ask questions, and their consent was documented by the study facilitator before the focus group began.

Focus group questions were designed to elicit feedback about patient experiences with the diagnostic process, perceptions of diagnostic communication, and on the note sheet's clarity, usability, and perceived value. Focus group questions included: “What does the term ‘diagnostic error’ mean to you?”; “How do doctors include you in the diagnostic process?”; “Which aspects of the note sheet were challenging for you to complete?”; “How do you think this note sheet could be improved so that you, or another patient, could use this more readily?”; and “What advice would you give a patient who was seeing this note sheet for the first time?” Patient focus group discussions were audio recorded, transcribed, and reviewed for accuracy by the research team. All transcripts were de-identified and summarized by key themes by two reviewers. Key themes were generated by a research assistant and the project lead separately, then reviewed and compared in research team meetings. These summarized themes were then presented to the focus group participants to confirm accuracy and seek additional feedback and clarification. Table 1 presents a summary of the modifications made to the existing AHRQ “Be the Expert on You” note sheet from feedback in patient focus groups. Given the minor changes made to the tool itself, we planned to administer only the modified tool to all patients, believing that the modified tool could meet the diagnostic communication needs of both older adults and the general adult population.

**Table 1:** Summary of modifications made to AHRQ's “Be the Expert on You” note sheet.

Change Made	Rationale
Add “pain” as an option for the question “why are you here today?”	Focus group members felt it was important to include, as it is a common motivation for them to seek care. Participant comments: P1: “That first time when the doctor comes in, it matters. They need to stick with you, asking, ‘What can I do for you today? Are you in pain?’ The only thing I would add is, ‘Are you in pain? Where, and what is the level of your pain? It's important to me.’”
Replace “how does [a change in how you are feeling since your last visit] affect you?” with “how does [a change in how you are feeling] make you feel?”	Focus group members expressed concerns about being able to identify and describe appropriate effects of clinical changes to share with providers. They felt the modified language made it more inviting and comfortable for them to express their own perspectives. Participant comments: P4: “Is it more effective for me to fill it out from my perspective or is it more effective for me to fill it out from his [the provider's] perspective?”.
Replace “what are you worried about” with “is there anything else going on?”	Focus group members felt that patients might not feel comfortable writing and admitting being “worried” about something. They felt patients should be encouraged to express their thoughts, as it could be a potentially important aspect of their health story that affects diagnosis. Participant comments: P3: “Very few people our age go volunteer anything. Nobody's going to admit ‘What are you worried about?’ or tell you ‘I'm worried about depression.’ It's a bit too direct, maybe soften it to ‘What else is going on?’”
Added a note-taking space for patients to use during their appointment	Focus group members requested space on the note sheet to be able to take notes and organize their thoughts, and to refer back on. Participant comments: P6: “And if you want to make a note to yourself, you want to have a space about your impression or afterthought of your visit.” P2: “I personally will put down what I discussed with the doctor. There should be some kind of comments area. ‘Where is a spot if you want to put the doctor's comments down?’ There should be space available for the remarks.”
Added a checklist of diagnostic- and health-related tasks that need to be completed in the appointment (e.g., laboratory testing, vaccination, obtain specialty referral information, speak to clinical educator or social worker, get a work or school note)	Focus group members, staff, and physicians described the need for a checklist and plan of action to ensure that diagnostic-related tasks are completed. Participant comments: P5: “There's something missing, [there needs to be] the process for the front desk. The front desk needs to be able to look at this and see ‘Okay, you need to reschedule an appointment’…too many times the doctor has left and I'm not clear on what to do.”
Added information for front desk to make follow-up appointments (e.g., visit type, follow-up time period in weeks, provider type, double-booking allowed)	Physicians, nurses, and staff described the need for this information to be added to improve current clinic workflows.

### 2.4 Phase 2: provider, nurse, and staff training for diagnostic communication and note sheet Use

A physician champion was responsible for leading implementation and was routinely updated on patient discussions and changes to the note sheet throughout the focus group phase. Because of the key roles that other patient-facing clinicians and staff members play in diagnostic safety efforts (1, 26), all practice physicians, nurses, and staff members (i.e., front desk, administrative, and patient navigation staff) received training from the physician champion about broad diagnostic safety concepts and the use of the note sheet in their new workflow. Physicians, nurses, and staff had not been previously familiar with the AHRQ note sheet or other diagnostic safety concepts. Training consisted of an introduction of the Toolkit Infographic and One-Page Handout for Staff Training (10) through in-service sessions and daily team huddles. In these sessions, clinicians and staff gave additional feedback on diagnostic safety concerns within the practice, explaining that patients occasionally failed to schedule follow-up appointments or complete care activities (e.g., laboratory tests, specialist referrals) which impacted the diagnostic process. They suggested adding a section to the note sheet to address clinical care needs prior to patient check-out to mitigate these concerns and better integrate the note sheet in the clinical workflow.

### 2.5 Phase 3: practice-wide intervention implementation in 3 phased PDCA cycles

This was the first diagnostic safety intervention to be implemented by physicians, nurses, and staff in this practice, and also the first time a communication tool was introduced to patients at this practice. Based on physician and practice leadership feedback, implementation of the modified “Be the Expert on You” note sheet in clinical practice occurred in three stages over the course of 6 months and included three iterative PDCA cycles to ensure that a continuous loop of workflow improvement effectively occurred (27). The PDCA cycle is a continuous loop of planning, doing, checking (or studying), and acting where testing of quality improvement measures occurs on a small scale before updating procedures and working practices (28).

We had initially planned for front desk staff to introduce the note sheet and allow patients to complete it while they were in the waiting room. However, after speaking with patients and care team members, we decided to have the note sheet introduced by nurses during the triage process. Patients expressed a desire to have the note sheet introduced by a health professional, citing the need to be encouraged to share their perspective in the context of diagnostic safety. Physicians and staff expressed the need for the note sheet to be included in the workflow in a way that accommodated concerns about high front desk staff turnover and clinic workflows.

We worked with our patient participants and physician champion to develop an adapted script from the One-Page Handout for Staff Training (10), intended for nurses to introduce the modified note sheet to patients. Nurses were provided with the script by physicians at the start of their workday, and used the script to verbally introduce the note sheet to all English-speaking adult patients after their vital signs and intake were completed. Patients were asked to fill out the note sheet while waiting to see the physician. Patients and physicians were instructed to review the note sheet together and use it to guide the health history communication. Patients were then offered the opportunity to have a scanned copy of the note sheet for their own records and asked to leave the completed note sheet in a box after checkout. All patients were invited to complete a post-appointment survey to provide feedback about using the note sheet during their appointments.

To gather feedback on the implementation process across multiple PDCA cycles, practice leadership suggested a phased approach. Initially, the physician champion piloted the modified note sheet with a designated group of patients and nursing staff over a two-week period. Feedback from the first PDCA cycle was then discussed in staff meetings, leading to minor revisions of the note sheet and workflow processes to address staff concerns and questions. These included adjustments to font size and wording, the addition of future appointment and testing reminders to the note sheet, modifications to checkout procedures, and improvements in the process of scanning and recording completed note sheets in patients' charts. The second PDCA cycle expanded the implementation to include six additional physicians and six nurses over a three-month period. Feedback from patients, physicians, nurses, and staff during this phase was reviewed and used to refine the implementation plan for the subsequent practice-wide rollout. Nurses confirmed that the script was appropriate and could be implemented within their workflows, and staff felt satisfied with the modifications to the patient checkout process and checklist of care activities provided by the diagnostic communication tool. In the final PDCA cycle, the modified note sheet was implemented across the entire practice, involving all physicians and staff over a three-month period.

### 2.6 Data collection

Surveys consisting of closed- and open-ended questions were developed by the study team to collect information about patients', physicians', and staff members' experiences with diagnostic communication throughout the project. No exclusion criteria were applied to patients during intervention implementation.

#### 2.6.1 Physician, nurse, and staff surveys (pre- and post-)

To gain a better understanding of both physician and other healthcare team members' perspectives, surveys were distributed to all physicians and nurses and staff before and after implementation of the modified “Be the Expert on You” note sheet. A 5-point Likert scale was used to assess respondents' level of agreement with the following statements: “*My patients come to their appointments prepared”, “My patients effectively communicate their health needs”, “My patients share their health stories in an efficient manner”, “My patients are helpful partners in the diagnostic process”, and “My patients are organized with the important health information needed for me to make a diagnosis.”*

#### 2.6.2 Patient surveys and note sheet utilization and completion rates

During the 6-month implementation period, surveys were distributed to gather insights on usability and satisfaction from the patient perspective. Responses remained anonymous and served as consent to participate in the project. Patients were asked to rate their level of agreement on a 5-point Likert scale to the following statements: “*The provider listened to me carefully during my visit”, “The provider addressed my main concerns”, “The ‘Be the Expert on You’ note sheet helped me to organized my thoughts”, “The ‘Be the Expert on You’ note sheet helped my communication with my provider”, “I am satisfied with this note sheet”, and “I would recommend this note sheet to other patients.”* Open-ended questions were included to encourage patients to describe any perceived challenges and/or benefits of using the modified note sheet during their visit. Demographic information (e.g., age, gender, race/ethnicity) was also collected.

To improve survey completion rates, patients were offered the opportunity to participate in a raffle for a $100 gift card by including their name and contact information (email or phone) at the end of the survey.

## 3 Analysis

Survey responses were collected by the study team at each phase of implementation, recorded into an Excel spreadsheet, and stored in a cloud-based content management platform for analysis. Note sheet utilization rate (i.e., number of note sheets collected divided by numbers of patients seen) and average note sheet completion rates (i.e., percentage of note sheet that was completed by patients) were also evaluated. Descriptive statistics were completed to describe patient, physician, nurse and staff survey results. We employed means for continuous variables and frequencies and percentages for categorical variables. All analyses were conducted using STATA version 14.2 (29).

## 4 Results

### 4.1 Physician, nurse, and staff surveys (pre- and post-)

Fifteen physicians and 13 nurses and staff members responded to the pre-intervention survey; 12 physicians and 8 nurses and staff members responded to the post-intervention survey. We observed little change in the proportion of physicians who agreed with the statement, “my patients come to their appointments prepared” pre- and post- intervention; fewer staff agreed with the statement that patients came to appointments prepared after the note sheet intervention. Before implementing the “Be the Expert on You” note sheet, no physicians and only 1 nurse or staff member (3.6% of the total sample) agreed with the statement, “my patients are organized with the important health information needed for me to make a diagnosis”; after the intervention, 2 physicians and 3 nurses and staff members (25.0% of the total sample) agreed with this statement. Prior to the note sheet implementation, only 1 physician and 2 nurses and staff members (10.7% of the total sample) agreed with the statement, “patients share their health stories in an efficient manner”; after implementation, more than half of physicians and nurses and staff members (60.0% of the total sample) agreed with this statement. Additionally, after implementing the note sheet, more physicians and staff collectively agreed with the statement, “my patients effectively communicate their health needs” (75.0% vs. 42.9%). In the physician respondent subsample, 83.4% of physician respondents agreed with the statement, “my patients effectively communicate their health needs” after the note sheet implementation, compared to 33.3% of physician respondents before implementation. Table 2 presents the responses from our physician and nurse and staff subsamples.

**Table 2:** Physician, nurses, and staff pre- and post-intervention responses.

Statement	**Physician Responses, *n* (%)** Pre-intervention, *n* = 15 Post-intervention, *n* = 12			**Nurses and Staff Responses, *n* (%)** Pre-intervention, *n* = 13 Post-intervention, *n* = 8		
	Strongly Agree or Agree	Neither Agree nor Disagree	Strongly Disagree or Disagree	Strongly Agree or Agree	Neither Agree nor Disagree	Strongly Disagree or Disagree
My patients come to their appointments prepared.	4 (26.7)	5 (33.3)	7 (40.0)	3 (23.1)	7 (46.2)	4 (30.8)
	4 (33.3)	3 (25.0)	5 (41.7)	0 (0)	4 (50.0)	4 (50.0)
My patients are organized with the health information needed for me to make a diagnosis.	0 (0)	7 (40.0)	3 (60.0)	1 (7.7)	7 (53.8)	5 (38.5)
	2 (16.7)	2 (33.3)	6 (50.0)	3 (37.5)	4 (50.0)	0 (0)^a^
My patients effectively communicate their health needs.	5 (33.3)	5 (33.3)	5 (33.3)	7 (53.8)	5 (38.5)	1 (7.7)
	10 (83.4)	2 (16.6)	0 (0)	5 (62.5)	2 (25.0)	1 (12.5)
My patients share their health stories in an efficient manner.	1 (6.7)	4 (26.7)	10 (66.6)	2 (15.4)	8 (61.5)	3 (23.1)
	5 (41.7)	3 (25.0)	4 (33.3)	7 (87.5)	1 (12.5)	0 (0)
My patients are helpful partners in the diagnostic process.	6 (40.0)	8 (53.3)	1 (6.7)	3 (23.1)	9 (69.2)	1 (7.7)
	6 (50.0)	6 (50.0)	5 (41.7)	3 (37.5)	5 (62.5)	0 (0)

Practice staff includes front desk and administrative staff, and patient navigators.

^a^ Missing a survey response for this item.

### 4.2 Note sheet utilization and completion rates

We evaluated note sheet utilization and completion rates throughout the three phrases of the implementation period. A total of 143 patients completed at least one section of the “Be the Expert on You” note sheet. In the first phase of implementation (i.e., physician champion only), all 16 patients seen in clinic by the physician (100%) completed the note sheet in the 2-week period. In the second phase (i.e., 7 physicians), 39 of the 354 (11.0%) patients seen completed a note sheet in the 4-week period. During the 6-week implementation period occurring practice-wide, 88 of the 1,100 (8.0%) patients seen in clinic completed a note sheet.

Of those 143 patients who engaged with the note sheet in some capacity, 100 patients (69.9%) completed the note sheet in its entirety (i.e., completed each of the five questions). We found that all of these patients answered the first question (“Why are you here today?”) and third question (“Have you seen anyone else about your health?”). The lowest percentage of patients (80.4%) responded to the last question of the note sheet, “Is there anything else going on?”

### 4.3 Older adult patient surveys and feedback

We received 120 patient survey responses, with 31 surveys from patients 65 years of age or older (21.7%). Survey participants were predominantly women (71.7%) and Black or African American (81.7%) (see Table 3).

**Table 3:** Patient survey demographics (*n* = 120).

Variable	*n* (%)
Age (in years)	
21 or younger	7 (5.8)
22–34	11 (9.2)
35–44	19 (15.8)
45–54	20 (16.7)
55–64	26 (21.7)
65–74	26 (21.7)
75 and older	5 (4.2)
*Missing*	6 (5.0)
Gender	
Man	28 (23.3)
Woman	86 (71.7)
*Missing*	6 (5.0)
Race	
American Indian or Alaska Native	0 (0.0)
Asian or Pacific Islander	2 (1.7)
Black or African American	98 (81.7)
Other or multiple	6 (5.0)
White	5 (4.2)
*Missing*	9 (7.5)

A majority of older adult patients who responded to survey questions (*n* = 31) agreed or strongly agreed that the “Be the Expert on You” note sheet helped them to organize their thoughts (71.0%) and helped their communication with their physician (77.4%). Most older adult patients reported being satisfied with the note sheet (80.6%) and would recommend the note sheet to other patients (77.4%). More than three-quarters of older adult patients who used the note sheet felt their provider listened to them carefully during their visit (77.5%) and addressed their main concerns (80.6%). The responses from the older adult subsample, as well as from the broader patient sample, are presented in Table 4. With the exception of the item regarding recommending the note sheet to other patients, we found that the responses to all other items were similar between the older adult subsample and the full patient sample, with differences within 10%.

**Table 4:** Older adult (*n* = 31) and all patient (*n* = 120) responses.

Question	Older adult responses, *n* (%) All patient responses, *n* (%)					
	Strongly Agree	Agree	Neither Agree nor Disagree	Disagree	Strongly Disagree	Missing
The “Be the Expert on You” note sheet helped me to organize my thoughts.	14 (45.2)	8 (25.8)	4 (12.9)	0 (0)	3 (9.7)	2 (6.5)
	60 (50.0)	34 (28.3)	12 (10.0)	0 (0)	10 (8.3)	4 (3.3)
The “Be the Expert on You” note sheet helped my communication with my provider.	15 (48.4)	9 (29.0)	3 (9.7)	0 (0)	2 (6.5)	2 (6.5)
	64 (53.3)	29 (24.2)	14 (11.7)	0 (0)	9 (7.5)	4 (3.3)
The provider listened to me carefully during my visit.	22 (71.0)	2 (6.5)	3 (9.7)	0 (0)	2 (6.5)	2 (6.5)
	88 (73.3)	17 (14.2)	3 (2.5)	0 (0)	9 (7.5)	3 (2.5)
The provider addressed my main concerns.	20 (64.5)	5 (16.1)	1 (3.2)	0 (0)	2 (6.5)	3 (9.7)
	87 (72.5)	17 (14.2)	2 (1.7)	0 (0	9 (7.5)	5 (4.2)
I am satisfied with this note sheet.	17 (54.8)	8 (25.8)	2 (6.5)	0 (0)	3 (9.7)	1 (3.2)
	68 (56.7)	30 (25.0)	9 (7.5)	0 (0)	10 (8.3)	3 (2.5)
I would recommend this note sheet to other patients.	20 (64.5)	4 (12.9)	2 (6.5)	0 (0)	4 (12.9)	1 (3.2)
	71 (59.2)	28 (23.3)	6 (5.0)	0 (0)	12 (10.0)	3 (2.5)

We asked all patients to describe any challenges utilizing the note sheet and to identify ways that the note sheet helped them with their health visit. Of the 35 free-text comments received related to challenges using the note sheet, 30 patients (85.7%) responded “none” or “n/a”. One patient reported they had “nothing to write on” and that a clipboard might be helpful; two patients reported lost information or information-related challenges. The remaining two comments described appointment details rather than the note sheet itself.

Thirty-two patients responded to the question about how the note sheet helped with their health visit. Several patients (*n* = 6) stated the note sheet helped them focus, for example: “It helped me stay focused on what is ailing me and tell the doctor without rambling.” Other patients stated that the note sheet helped them communicate better (*n* = 4) and helped to prepare or get organized prior to meeting with the physician (*n* = 4): “It helped me think out what I needed to ask my physician.” A few patients (*n* = 3) expressed that the note sheet helped the physician with the diagnosis, “It allowed the doctor to immediately identify my issues,” and one patient wrote that the note sheet “Shows that the staff is concerned about its patients.”

## 5 Discussion

Quality improvement can benefit from early and meaningful integration of patients and families in all aspects of the process. While definitions of patient engagement vary widely, strategies that foster a partnership and shared leadership between healthcare providers and patients are generally considered the highest level of engagement. These strategies have the potential to yield better, more patient-centered outcomes (30). However, codesigning with patients is a strategy that is often absent or undefined in the existing literature, and particularly with input from patients of different ethnic backgrounds, age groups, and disability statuses (31). In our study, patient focus groups provided a meaningful opportunity for research and clinical teams to introduce diagnostic safety topics to older adult patients and engage them in codesigning strategies to improve diagnostic communication. As older adult patients are often underrepresented in patient safety and quality initiatives, they may require additional support and accommodations for meaningful involvement in codesign. Our experience highlighted the desire of older adult patients to be informed and encouraged to take on active roles in their own medical care, and demonstrates the positive outcomes of a relatively simple, low-cost intervention on older adult patient satisfaction and communication effectiveness.

In response to feedback from patient focus group sessions, several changes were made to the original note sheet wording and structure to improve older adult engagement and uptake. Older adult patients described the need for the note sheet to be introduced by a healthcare team member to understand their roles and be encouraged to participate in diagnostic safety initiatives. Patients who used the note sheet generally found it easy to use, helpful for communicating with their physicians, and would recommend its use to other patients. Patients described few challenges or concerns about using the note sheet.

Physicians, nurses, and staff members were generally satisfied with the note sheet, described few challenges to using it in practice, and agreed favorably about its use in improving information gathering and diagnostic communication. Somewhat unexpectedly, in our post-survey, we noted that no nurses and staff agreed with the statement that patients came prepared to their appointments. This lack of agreement may have been due to our decision for nurses to introduce and provide the note sheet to patients immediately prior to the physician encounter (during the triage process), rather than sharing the note sheet with patients prior to their appointment altogether. Alternatively, this finding may also represent an increased awareness of the role of patients in the diagnostic process and thus, an increase in expectations for patients to prepare and share their health stories more effectively.

Despite increased research and attention on patient engagement interventions, few studies have assessed the outcomes and impacts of various patient engagement interventions on quality outcomes (32), and particularly diagnostic quality. To our knowledge, this is the first study to determine the appropriateness of a diagnostic communication tool for older adults and to evaluate the uptake of AHRQ's “Be the Expert on You” note sheet in a primary care setting. We found uptake of the note sheet across all patients to be less than 10%; however, our implementation occurred in tiered phases and was implemented practice-wide for only 3 months to accommodate the overall one-year project time constraint. The proportion of completed note sheets to patients seen in clinic seems low; however, we observed a consistent and promising increase of completed note sheets over the short time period and positive feedback from patients, physicians, and staff. It is worth noting that implementation occurred despite several competing practice priorities and responses to the COVID-19 pandemic (e.g., high front desk staff turnover, building reconstruction, and blocked patient rooms), was relatively simple and required minimal workflow changes or additional resources, and that physicians and staff expressed an interest in continuing its use after our project period. Future efforts utilizing this tool should consider research studies that evaluate the uptake of the note sheet across longer implementation periods, deploy study designs that include comparison groups and/or can better ascertain the relationship between the intervention and diagnostic outcomes, and develop ways to accurately embed and/or track the patient note sheet across workflows and using electronic health record or technologies.

We found that nearly all patients who used the note sheet felt that their physicians listened to them carefully and addressed their main concerns, with more than 70% strongly agreeing with these statements. However, we were unable to compare this finding with the response rates of patients not utilizing the note sheet and there is a lack of comparable data around the “Be the Expert on You” note sheet and other diagnostic communication interventions in the existing literature. Future studies can build on our preliminary findings to examine whether the “Be the Expert on You” note sheet improves not only diagnostic communication but also patients' general experiences around communication in care appointments. Tailoring interventions to patients' preferences is necessary, particularly for historically marginalized patient groups, to ensure satisfactory experiences and help physicians to be more attuned to specific cultural and micro-cultural factors during medical encounters (33). Employing a patient co-design approach to adapt existing communication interventions may be an effective and relatively simple way to improve patients' communication experiences with their primary care physicians. Further research is needed to test the relationship between patients using the note sheet and their perceptions of being listened to and having their concerns addressed, particularly among ethnic minority and historically disadvantaged groups.

Our study has several strengths. Although the AHRQ “Be the Expert on You” note sheet was developed with patient partners and from patient input, additional changes and considerations were needed to make this more appropriate for our older adult population. Second, it demonstrates the feasibility of recruiting and engaging patient partners and implementing an intervention with several PDCA cycles as a one-year quality improvement initiative to achieve end user buy-in and engagement.

Despite these strengths, the results should be interpreted in light of a number of limitations. Because our study was conducted in one family practice, the generalizability of our findings is limited. Because this was intended to be a quality improvement initiative, we focused on implementation and did not have a comparison group available or evaluate diagnostic outcomes such as reported errors, time to diagnosis, or diagnostic accuracy. The small physician and staff sample size (both less than 20) limited our ability to perform nonparametric statistical tests to compare survey responses before and after the note sheet implementation. We did not have information available on non-respondents and therefore were unable to examine patient-level factors likely to contribute to engagement and the extent or impact of response bias. Finally, and despite recruitment efforts to include them, caregivers and family members of older adult patients were not involved in our focus groups. Caregivers are critical historians and messengers about any acute changes in older adults' symptoms, and there are few formal channels for caregivers to share information that could be essential to improving diagnosis. Future research considering the roles of caregivers and ways to better involve them in patient-facing interventions to improve diagnostic quality and other quality initiatives are needed.

## Data Availability

The datasets presented in this article are not readily available because with patient focus group transcripts, the small group size could compromise participant confidentiality and the proprietary nature of the information. De-identified excerpts relevant to the study's conclusions are available from the authors upon reasonable request, subject to ethical approval and confidentiality agreements.
